# The Contradictions of Telehealth User Experience in Chronic Obstructive Pulmonary Disease (COPD): A Qualitative Meta-Synthesis

**DOI:** 10.1371/journal.pone.0139561

**Published:** 2015-10-14

**Authors:** Lisa Brunton, Peter Bower, Caroline Sanders

**Affiliations:** 1 NIHR School for Primary Care Research, Manchester Academic Health Science Centre, University of Manchester, Manchester, United Kingdom; 2 NIHR Greater Manchester Primary Care Patient Safety Translational Research Centre, Manchester Academic Health Science Centre, University of Manchester, Manchester, United Kingdom; The University of Queensland, AUSTRALIA

## Abstract

**Objective:**

As the global burden of chronic disease rises, policy makers are showing a strong interest in adopting telehealth technologies for use in long term condition management, including COPD. However, there remain barriers to its implementation and sustained use. To date, there has been limited qualitative investigation into how users (both patients/carers and staff) perceive and experience the technology. We aimed to systematically review and synthesise the findings from qualitative studies that investigated user perspectives and experiences of telehealth in COPD management, in order to identify factors which may impact on uptake.

**Method:**

Systematic review and meta-synthesis of published qualitative studies of user (patients, their carers and clinicians) experience of telehealth technologies for the management of Chronic Obstructive Pulmonary Disease. ASSIA, CINAHL, Embase, Medline, PsychInfo and Web of Knowledge databases were searched up to October 2014. Reference lists of included studies and reference lists of key papers were also searched. Quality appraisal was guided by an adapted version of the CASP qualitative appraisal tool.

**Findings:**

705 references (after duplicates removed) were identified and 10 papers, relating to 7 studies were included in the review. Most authors of included studies had identified both positive and negative experiences of telehealth use in the management of COPD. Through a line of argument synthesis we were able to derive new insights from the data to identify three overarching themes that have the ability to either impede or promote positive user experience of telehealth in COPD: *the influence on moral dilemmas of help seeking*—(enables dependency *or* self-care); *transforming interactions* (increases risk *or* reassurance) and *reconfiguration of ‘work’ practices* (causes burden *or* empowerment).

**Conclusion:**

Findings from this meta-synthesis have implications for the future design and implementation of telehealth services. Future research needs to include potential users at an earlier stage of telehealth/service development.

## Introduction

The rise of the global burden of chronic disease, such as COPD, has led to a shift in health policy to target the prevention and management of chronic disease [1]. Policy makers have shown a strong interest in adopting remote monitoring via information and communication (ICT) technologies (termed telemonitoring, telehealth, telecare, telemedicine, or telehealthcare) to reduce the demand for expensive hospital admissions whilst helping people to live independently for longer [2]. However, despite such policy drives, remote monitoring has largely failed to be successfully integrated into routine healthcare [3, 4]. This article focuses on telehealth technologies which we define as those which (as a minimum) enable data, such as oxygen saturation levels, blood pressure and pulse, and other information to be remotely exchanged between a patient and health professional as part of their overall management of chronic disease [5]. We are concerned with understanding the perceptions of the users of telehealth (people with COPD, their carers and health professionals) to understand the factors which may impact on its uptake and sustained use.

It is estimated that 65 million people have moderate to severe COPD worldwide [6] and it is set to become the third leading cause of death by 2030 and the fifth leading cause of disability by 2020 [7, 8]. Patients experience exacerbations of their condition which can lead to hospitalisation and, despite the drive for COPD to be managed within the primary care setting, it is the second biggest cause of emergency admission to hospital [9]. Interventions are needed to effectively manage COPD to reduce the burden on health care resources and individuals alike.

A number of quantitative systematic reviews have been conducted to assess the effectiveness of telehealth in COPD [4, 10, 11] and these have showed mixed results. For example, Bolton et al., 2011 [4] determined there was inadequate evidence to support the widespread implementation of telehealth in COPD due to the small, low quality and heterogeneous studies conducted. A Cochrane review [11] indicated that telehealth technologies had the potential to improve quality of life, reduce COPD exacerbations, emergency and hospital visits compared to usual care. This supports results from a previous systematic review that found telehealth (both home telemonitoring and telephone support) reduced emergency and hospital visits [10]. Each systematic review used different definitions of telehealth and therefore different studies (with different interventions) were included, so there is difficulty in pinpointing the successful ‘active ingredient’ in the telehealth interventions assessed.

Telehealth interventions often include many components and can be described as ‘complex interventions’ [12]. However, evaluations of telehealth do not routinely follow the MRC framework for complex interventions: often foregoing the crucial early phases (theoretical work and modelling how the intervention works) and heading straight into later phases (pilot trials or RCTs) [11]. Since user involvement is a key component of the modelling phase, this means that the users’ voice is often ignored in the process of such technology development [13]. However, users need to be involved in all processes of technology and service development, if successful implementation is to occur [14].

Whilst a number of qualitative studies have been conducted that give some insight into how users perceive this technology, to date these studies have not been systematically synthesised. Conducting a synthesis of qualitative studies will give a deeper insight into the perspectives of clinicians and patients in the use of telehealth by further developing the theories and concepts that individual reading of studies cannot bring [15].

The aims of the study were to:

1. Systematically search the literature to identify relevant qualitative studies that explored user experience of telehealth in COPD
2. Conduct a meta-synthesis to identify shared themes in user experience across studies and gain new insights from synthesising the data
3. Discuss how findings can contribute to the design of new or the refinement of existing telehealth technologies and services

## Methods

### Selection criteria

Only full text, peer reviewed published qualitative studies were included in the review; therefore, we excluded unpublished studies, dissertations, book chapters and conference abstracts. We also excluded studies which did not support their qualitative findings with verbatim quotes/observational field notes as they do not enable reviewers to judge authors’ interpretation of the data or the quality of analysis undertaken [16]. We included studies which explored users’ experience of telehealth technologies and excluded studies which reported only the views of people who did not have a direct role in providing patient care (such as commissioners or technology suppliers); studies whose interventions did not correspond to our definition of telehealth (see introduction) were excluded (e.g. telephone consultations between patient and their health care provider or online internet self-help programmes). Due to resource constraints, we only included studies that were published in the English language.

### Search strategy

To simplify the reporting of the search strategy, we outline 3 stages of searching that we carried out. In reality, the searching process was not a linear process prior to synthesis, rather, an iterative process of searching and synthesising occurred. Our knowledge of the literature from conducting previous research in the area, together with initial searches started off the synthesis process; we then conducted more searches and added papers as they were identified (but all 10 included papers were captured in the final searches reported below).

The search strategy was developed by all authors and following advice from a librarian with expertise in database searching. 1. Test searches were carried out by LB to identify key words and titles, after which we increased the number of telehealth search terms and removed the ‘qualitative research filter’ [17] to optimise sensitivity of the search and the number of studies identified. 2. LB searched 6 electronic databases with the finalised search terms from their inception to October 2014: ASSIA; CINAHL; Embase (Ovid); Medline (Ovid); PsychInfo (Ovid); Web of Knowledge; these were chosen because they represent the areas of nursing, medicine, social sciences and psychology [18]. The finalised key search terms (and their variants) were grouped into two categories: ‘telehealth’ (intervention) and COPD (population). Each category consisted of a mixture of medical subject headings (MeSH) and free text terms which were adapted to suit each individual database (see S1 File for Ovid Medline search strategy). 3. LB and CS manually searched the reference lists of included studies and key papers. Searching stopped when the included studies enabled us to sufficiently develop the concepts and theory to create the interpretive synthesis [19].

### Studies screening method

All search results were entered into Endnote, a bibliographic database, and duplicate references were removed before screening. LB independently assessed titles and abstracts for inclusion; ten percent of retrievals were reviewed by CS. Full text articles were retrieved for further scrutiny when a title and abstract appeared to meet inclusion criteria; where inclusion of a paper was judged to be equivocal, CS independently assessed for inclusion (n = 3). All papers judged to meet the inclusion criteria (n = 10) by LB were independently reviewed by CS before being included in the review.

### Assessment of quality

We appraised quality not to exclude studies but to ensure that we did not give too much weight to studies that were of lower quality [20]; hence, studies of lower quality were given less prominence when conducting the synthesis process. Excluding studies because of minor design errors can risk excluding important studies that could contribute valuable concepts to answering the question under study [19, 21]. Methodological quality of identified studies was assessed independently by two researchers (LB and AH-M) using the method outlined in the Masood et al., 2011 [22] paper. This uses an adapted version of the CASP qualitative appraisal tool [23] to answer up to 30 yes/no questions to assess the credibility, relevance and rigour of each paper (where evidence is present, a score of 1 is given; where no evidence is present, only partially present or unclear, a score of 0 is given). Not all questions are applicable to each paper so scores are calculated to produce a percentage in order to gauge how far each paper meets the CASP criteria. The researchers came together to discuss discrepancies in scores awarded; where discrepancies could not be resolved they were discussed with a third researcher (CS).

### Synthesis approach

Analysis was guided by the meta-ethnographic approach as first outlined by Noblit and Hare [24]; this approach was chosen for its ability to move beyond summarising the data (such as in a narrative approach) to achieve a more interpretive, conceptual explanation of the data. Most methods of synthesis are applicable to synthesising heterogeneous data; our synthesis is in keeping with others who have used a meta-ethnographic approach to synthesise studies that used disparate methodologies [25]. Whilst the included studies used different methodologies, these could be categorised as broadly thematic in terms of the endpoint of analysis which we could identify as secondary constructs for comparison.

Papers were independently read and re-read by LB and CS and first and second order constructs were extracted from the results, discussion and conclusion sections of the papers and entered into Nvivo 10 (computer assisted qualitative data analysis software package) in order to manage the data. First order constructs refer to participants’ verbatim quotes and second order constructs refer to the authors’ interpretations of the participants’ quotes, usually expressed as themes or categories within the papers. Third order constructs are the new interpretation or theory that is derived from synthesising first and second order constructs [20]. Constructs were independently reviewed by LB and CS to identify how they compared and contrasted across papers; this enabled us to identify how studies were related [26]. The second order constructs were further scrutinised by LB and CS and third order constructs were developed that summarised and incorporated the various concepts across studies; to enable this, LB extracted the first and second order constructs into a spreadsheet under the themes of our emerging 3^rd^ order constructs to see how studies translated into one another. Third order constructs were further refined through discussion at regular researcher meetings (with all authors) until consensus was reached.

In order to make sense of consistent themes and contradictions within the data (mainly stemming from the different perspectives of patients and health professionals), we developed a ‘line of argument’ synthesis. Essentially, this type of synthesis is concerned with inference; that is, it serves to build a ‘whole’ that makes sense of its parts [24]. This was achieved by first translating the studies into one another (as described above) and then developing a new interpretive context by exploring the similarities and differences identified within and across studies [24].

## Findings

### Quality appraisal

The quality scores are presented in Table 1 and S2 File. Overall, findings were clearly presented, and five papers met at least 76% of the CASP criteria; one study [27] met only 45% of CASP criteria and was considered to contribute less to the synthesis: this was due to low quality appraisal score, and thin description. Throughout the papers, there was scant reporting of reflexivity and limited reporting of ethical issues raised by the studies.

**Table 1:** Characteristics of included studies.

References (n = 10) Country setting	Research type	Aims	Sample Patients* (age, gender) health professionals (job role) *all patients received telehealth	Patient group	Telehealth intervention (include monitoring and frequency, extra features and duration)	Time point of data collection	Data collection method	Data analysis method	Quality Appraisal% of how far paper met CASP criteria
Williams et al. (2014)[34] Oxford, UK	Qualitative study nested within a pilot study	Explore patients’ expectations and experiences of using Mhealth telehealth application to support self-management of COPD	19 patients: 11 men/8 women, 50–85 years (mean 67)	People with moderate to very severe COPD	*Monitoring and frequency*: patients completed pulse rate, oxygen levels and symptom diary daily. Data reviewed at regular intervals by research nurse (not daily). *Extra features*: multimedia educational materials (video, text, images). *Duration*: 6 months	Twice (before telehealth use and after 6 months use)	Semi structured interviews	Grounded theory approach	83%
Dinesen et al. (2013)[32] Region of Northern Jutland, Denmark	Qualitative study nested within an RCT	What are COPD patients’ attitudes towards telerehabilitation as seen from a learning perspective?	22 patients: 8 men, 64 to 74 yrs (mean 69); 14 women, 45 to 81 yrs (mean 66) 26 Health professionals (6 GPs; 4 hospital nurses; 2 hospital Drs; 6 healthcare centre nurses; 8 district nurses)	People with severe to very severe COPD	*Monitoring and frequency*: Health care professionals assessed patients' readings (blood pressure, oxygen levels, peak flow, weight) on the agreed days of self-monitoring and contacted patients. At onset of exacerbation, daily contact maintained between patient and healthcare professional. *Extra features*: telehealth part of pulmonary rehab programme. On agreed days, health professionals rang patients to discuss their health and offer advice. Patients could connect with other patients via web portal; exercise via games console. *Duration*: 16 weeks	Patients: 3 times (before, during and post telehealth use) Health professionals: after supervising patients’ entry into trial	Patients: Semi structured interviews plus participant observation Health professionals: semi structured interviews	Thematic analysis	79%
Fairbrother et al. (2013)[28] Lothian, Scotland, UK	Qualitative study nested within an RCT	Explore patients’ and professionals’ views on self-management in the context of telemonitoring in COPD	38 patients: 18 men/20 women, 44 to 85 yrs (mean 67.5) 32 professionals (included primary/secondary care nurses with several months’ experience of delivering telehealth service, COPD trial research nurses, GPs -some who declined trial involvement, service managers, information technology suppliers, support staff)	People with moderate to severe COPD	*Monitoring and frequency*: Monday to Friday telehealth service; weekday daily monitoring of symptom questionnaire and oxygen levels; weekly monitoring of weight and FEV1. Patient contacted if abnormal results or if no data submitted. *Extra features*: video link for remote consultations (but only used twice during study due to poor connection). Patients given (hard copy) self-management action plan and emergency supply of antibiotics and steroids. *Duration*: 1 calendar year	Patients: midway through telehealth use Professionals: after *several months’* experience of telehealth service	Semi structured interviews	Framework analysis	76%
Gale & Sultan (2013)[35] The Black Country, UK	Qualitative service evaluation	Understand how people with COPD experience and interact with telehealth to recognise how it incorporates into their everyday life and home space	N = 7 patients (5 male/2 female; mean age 66.9 years)	People with mild to very severe COPD Patients of a community respiratory service who had 3 or more COPD related hospital admissions in 12 month period and deemed able to use equipment	*Monitoring and frequency*: Monday to Friday telehealth service (as an addition to 12 hour, 7 day a week Community Respiratory service—CRS). Weekday daily monitoring of pulse, oxygen levels, blood pressure, weight and temperature. Patients contacted if abnormal results or if no data submitted. *Extra features*: none. *Duration*: 9 months	During telehealth use	‘Situated’ interviews (observation and interview)	Framework analysis	72%
Huniche et al. (2013) [33]See Dinesen et al., 2013 (same study)	See Dinesen et al., 2013 (same study)	Explore how COPD patients make use of readings during 16 weeks of self-monitoring in a telerehabilitation project	22 patients: 8 men, 64 to 74 yrs (mean 69); 14 women, 45 to 81 yrs (mean 66)	See Dinesen et al., 2013 (same study)	See Dinesen et al., 2013 (same study)	3 times (before, during and post telehealth use)	Semi structured interviews	Thematic analysis	69%
Fairbrother et al. (2012) [29] See Fairbrother et al., 2013 (same study)	See Fairbrother et al., 2013 (same study)	Investigate the views of patients and clinicians on the impact of telemonitoring service to continuity of care	See Fairbrother et al., 2013 (same study)	See Fairbrother et al., 2013 (same study)	See Fairbrother et al., 2013 (same study)	See Fairbrother et al., 2013 (same study)	See Fairbrother et al., 2013 (same study)	Framework analysis	83%
Ure et al. (2012)[36] Lothian, Scotland, UK	Qualitative study as part of pilot study evaluation	Assess the acceptability of telemonitoring service to patients and clinicians	20 patients: 13 men, 7 women, (mean age 68.9 yrs) 25 health professionals (4 GPS; 4 Practice Nurses; 2 hospital based respiratory nurses; 2 nurse managers; 2 physiotherapy managers; 3 physiotherapists; 2 non-clinical managers; 6 community nurse managers)	People with moderate to severe COPD	*Monitoring and frequency*: Monday to Friday telehealth service. Weekday daily symptom questionnaire and oxygen levels; weekly monitoring of weight and FEV1. Patients contacted if abnormal readings or if no data submitted. *Extra features*: Patients given (hard copy) self-management action plan and emergency supply of antibiotics and steroids *Duration*: 2 months	Patients: twice (before telehealth use and = />2months of telehealth use) Professionals: occurred at different time-points during service set up	semi structured interviews and 1 professional focus group	Techniques of grounded theory/thematic analysis	79%
Mair et al. (2008) [30] Liverpool, UK	Qualitative study nested within an RCT	Use the Normalisation Process to understand and interpret findings from a qualitative evaluation of RCT	N = 9 patients (no further info) N = 11 specialist respiratory nurses involved in telehealth trial	People experiencing an acute exacerbation of COPD	*Monitoring and duration*: blood pressure, temperature, oxygen levels (no information on frequency) *Extra features*: analogue video link for remote consultations. *Duration*: 2 weeks	No information	Semi-structured interviews	Framework analysis	62%
Horton (2008)[27] South East England, UK	Qualitative service evaluation	Evaluate telehealth	N = 3 healthcare professionals (no further details) involved in redesign of COPD service (included telehealth) and one key member from telehealth call centre + 6 case studies of patients receiving telehealth (no further details)	‘Older’ people following discharge from hospital after acute exacerbation of COPD	*Monitoring and frequency*: daily monitoring included oxygen levels, pulse and respiratory rate (no further info). Data transmitted to a local call centre, escalation procedure was set up so call centre operator would know what intervention would be appropriate. *Extra features*: none. *Duration*: 5 days	6 months post service implementation	Focus group + 6 case studies (chosen by health professionals) to examine key issues related to implementation of telehealth	Thematic analysis	45%
Hibbert et al. (2004) [31] See Mair et al., 2008 (same study)	See Mair et al., 2008 (same study)	Document the responses of specialist nurses using telehealth and identify key issues relating to its integration into routine care	N = 12 specialist respiratory nurses involved in telehealth trial	See Mair et al., 2008 (same study)	See Mair et al., 2008 (same study)	during set up and implementation of RCT	Participant observation	Using ethnographic principles’/constant comparison	62%

### Search results

**Fig 1:**
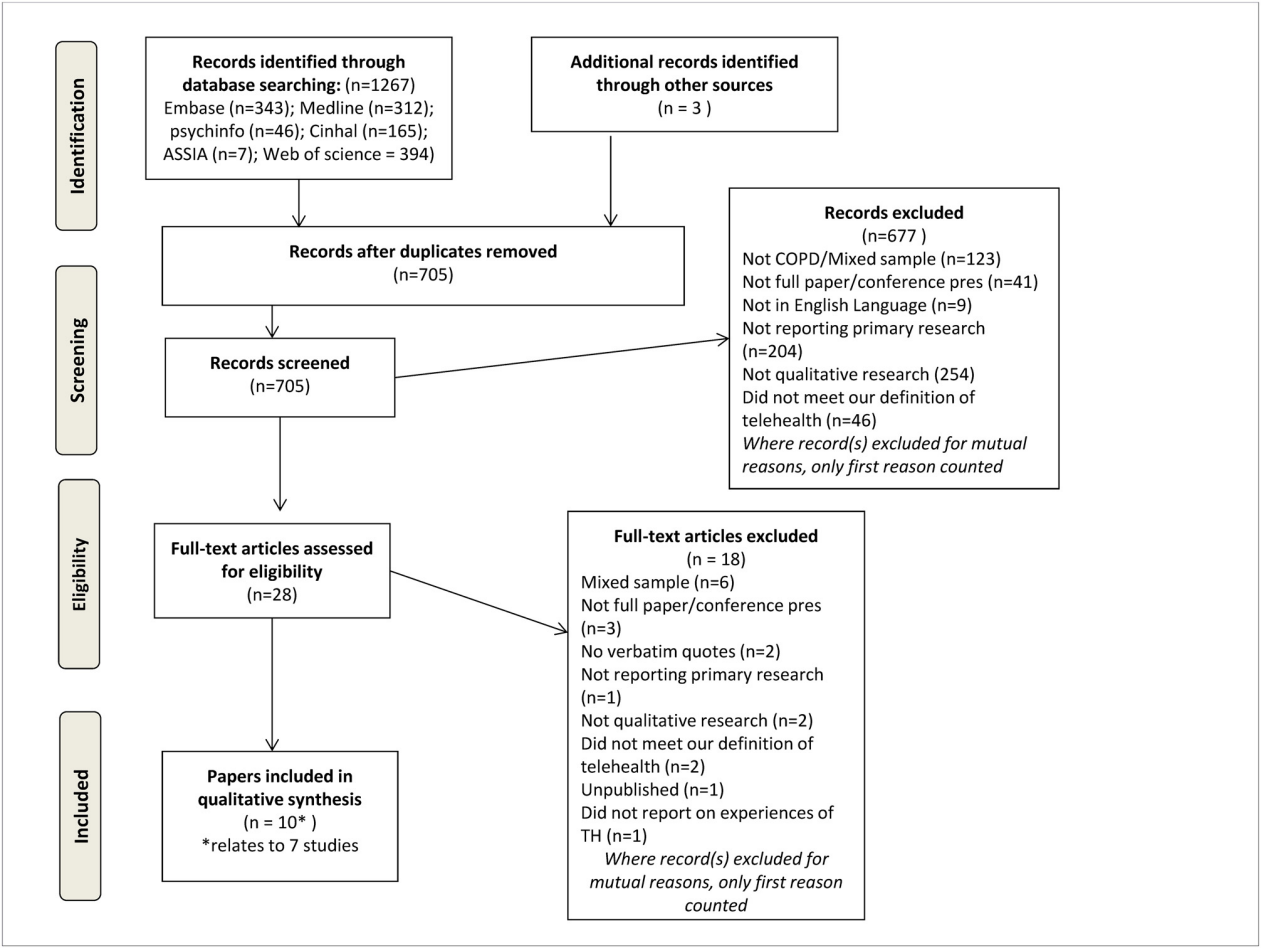
PRISMA flow diagram

Search outcomes are presented in the PRISMA flow diagram in Fig 1; the final searches retrieved 705 references (after duplicates removed), of which 10 papers were eligible for inclusion in the review. Table 2 details the papers selected for inclusion in the meta-synthesis. The ten papers relate to seven studies. Three studies had two publications reporting their qualitative findings: Fairbrother et al., 2013 and Fairbrother et al., 2012 [28, 29] utilised the same data sets to report the qualitative findings from a randomised controlled trial (RCT); two studies utilised overlapping datasets to report qualitative findings from an RCT (Mair et al., 2008 and Hibbert et al., 2004; Dinesen et al., 2013 and Huniche et al., 2013) [30–33]. Of the seven studies included, three were qualitative studies nested within RCTs of telehealth interventions; two were qualitative evaluation studies of small telehealth pilots, one was a nested qualitative study within a pilot study and one was a mixed methods (qualitative and quantitative) evaluation of a telehealth pilot study. Six of the studies were UK based and one was based in Denmark [32, 33].

**Table 2:** Examples of 1st, 2nd and 3rd order constructs.

Study	1^st^ order constructs	2^nd^ order constructs	3^rd^ order constructs
Dinesen et al., 2013	*“It has meant that I have been able to keep track*. *Now*, *I don’t know much about blood pressure…but the oxygen I have worked out”* Male patient, 3rd interview	Evaluating and keeping track of the state of the body [help] stay in control of the disease	Telehealth functions on a continuum between dependency/self-care. Telehealth takes away the moral dilemma of help-seeking: this can be perceived to increase dependency *or* enable self-care
Fairbrother et al., 2013	*“If you have a bad reading you’re not going to go out and do the gardening or go up and clean the bathroom or something”* Male, 72 years	many patients reported using telemonitoring data to validate their decision to self-medicate and/or to contact healthcare professionals	
	*I feel it reinforces a sick model for the patient*.* *.* *. *the patient would see themselves as very unwell on a daily basis because they’re constantly focussing on their disease state”*. Nurse, secondary care, ID24	[health care providers] expressed concern about creating dependence on the technology and/or practitioner support, particularly among patients with severe COPD	
Mair et al., 2008	*“*.* *.* *. *The main advantage is knowing that I can do it myself*, *if I see that it’s going down … bring a nurse out and they will come out”* Patient 74	[patients] felt that using the system… gave them more autonomy	
Williams et al., 2014	*“It’s a presence in the home*, *it encourages me to do what I call breathing exercises”* HA006	[telehealth] reminded patients of the need to engage in self-management…	
	*“they [respiratory nurses] tell me to do this when I’m not well and they [respiratory nurses] come and see me”* HA001	some patients perceived [telehealth] as less useful… those [patients] … appeared to be less engaged with managing their COPD	
Fairbrother et al., 2012	*“It makes you feel like somebody’s looking after you*. *If anything goes wrong*, *you can get in touch with them any time you want*.* *.* *. *you’ve got the confidence that they're going to get something done*” Male, 79 years	The service was extremely popular with patients who reported a sense of reassurance in having someone ‘watching over them’	Telehealth transforms interactions. This threatens positive user experience when it is perceived to impede clinical assessment or lead to a false sense of security; it enables positive user experience when it is perceived to increase social connectedness and reassurance
Gale & Sultan, 2013	*“I felt it was*, *I felt I was being monitored*, *I felt like a connection*, *to be honest*, *like you know*, *to the [CRS] team*” P4	they particularly valued the ‘connection’ that the telehealth brought …it was felt to be important that at the other end of the line there was a real person	
Mair et al., 2008	*“I think you physically need the patient in front of you…it’s when you start talking to them and getting more of a rapport with them that they open up and tell you things”* Nurse 6	…nurses believed that clinical interactions using the system were less likely to achieve an accurate and full clinical assessment. The patients… focused on the personal qualities of nurses	
	*“I thought I could have discussed anything they’re such nice people “* Patient 60		
Ure et al., 2012	*“I thought it would be more clear-cut*. *It didn’t occur to me it would be so difficult to work to a standard baseline*. *It’s a lot more complex than I thought it was going to be”* GP1	Clinicians…described the importance of interpreting the scores in context…[home] visits were made to clarify the reality of the individual clinical situation when the score breached the threshold	
Huniche et al., 2013	*“And my wife joins me every time and we discuss it and… it’s on Mondays and Fridays that we measure*. *We have a good time doing that*.*”* Male patient, 2^nd^ interview	Measuring values…becomes a shared practice between patients and relatives, thus securing and supporting their mutual involvement in the patient’s health	Telehealth reconfigures the nature of work for patients and health professionals. This is perceived as a burden when it increases patient anxiety, increases health professional workload or threatens professional identify; it is perceived to empower when it increases patients’ ability to self-care
Gale and Sultan, 2013	*“So my oxygen levels today were 95…95 is really good… So you think to yourself*, *‘I’m having a good day today’…Without this*, *I’m going to think*, *what’s my oxygen levels today*? *… Do I need to call the nurse*, *do I need to see the GP*?*”* P3	Patients were required to take their measurements and send them via the telehealth equipment to the CRS… they became confident over time with what numerical level was ‘normal’ or not and … understand and interpret their body through these numbers	
Fairbrother et al., 2012	*“… where community teams are involved with our COPD patients*, *we get a lot more contact*, *a lot more calls*, *a lot of those are not appropriate in our eyes*. *A lot of buck passing*. *I think potentially that’s quite damaging to patient care”*. ‘Usual care’ GP, ID9	While some GPs appreciated sharing communication about patients and working in partnership …others found the involvement of nonmedical community-based telemonitoring professionals intrusive, unwelcome and unhelpful	
Hibbert et al., 2004	*While they were setting up the equipment*, *the patient remarked ‘Aren’t you clever*!*’ and her husband added*, *‘On top of this you’re engineers’*	The technology was sometimes seen as undermining nurses’ professional security and credibility…telehealth…opened fundamental debates about professional values and status. The transporting and setting up of equipment were …inappropriate professional activities, with one nurse describing feeling like a ‘carpet fitter’	
Horton 2008	*I had to come down to the office…access the electronic care plan… We also spent a lot of time sorting out the telephone line*	Because of the inherent problems with the equipment, health care professionals reported that a lot of time was wasted	
	*in a way I think it made more work for the nurses after we’ve been in [after installation]*.		

Regarding the aims of the ten included papers, the majority (n = 4) aimed to explore the experiences of both patients and health professionals in the use of telehealth; three explored the experiences of patients only; two explored the experience of health professionals only, and one [32] explored patients’ attitudes to telerehabilitation from a health professional and patient perspective. Of the seven studies, four were using telehealth in the routine care of a patients’ long term condition (LTC); two were using telehealth in the care of patients following acute exacerbation of COPD, and one study was using telehealth as part of a telerehabilitation programme. This impacted on the length of time patients used telehealth equipment within the studies; length of telehealth use ranged from 5 days following acute exacerbation of COPD in the Horton study [27] to 1 calendar year in the Fairbrother et al. study [28, 29] study (which used telehealth for routine monitoring).

A distinction also exists between the types of telehealth interventions used within studies, as some studies used telehealth to only monitor patients’ physiological signs (such as BP, pulse, oxygen saturation levels, temperature etc.), whilst others utilised other technological features to further support self-management, such as video-links to facilitate remote consultations or getting patients to complete daily symptom questionnaires. Two studies had more comprehensive telehealth interventions to support self-management [32–34].

The interaction between health professionals and patients varied throughout the studies: some only contacted patients when their physiological or other monitoring data were abnormal or if they did not receive data, whilst other studies maintained more regular contact with patients regardless of their data results. Telehealth was an addition to usual care provided in the majority of studies; only two studies appeared to introduce telehealth intended as a new service delivery: in one study it was used as part of a COPD service redesign to integrate primary care, social care and a local call centre [27] and within an RCT, telehealth was compared to existing home visits in a nurse led service [30, 31]. When interpreting the findings from this meta-synthesis, the heterogeneity of studies therefore needs to be borne in mind.

The results are presented in relation to the third order constructs that emerged from the synthesis of the included studies (see Table 3), these are:

1. The influence of telehealth on moral dilemmas of help seeking
2. The transformation of interactions
3. Reconfiguring the nature of ‘work’

**Table 3:** 1st order constructs to show the self-care/dependency continuum in telehealth use.

towards dependency	towards self-care
*In a way it was a relief thinking that I should ignore my own thoughts on getting a doctor or something like that*. *This organisation was going to get hold of a doctor if their readings showed I needed a doctor* Ure 2012	*…if we had antibiotics or steroids*, *we could start them and then see on the Monday morning*, *you know*, *to assess what we had* Ure 2012
*I had one patient on the machine and he hasn’t been in hospital for a year…He accredits that to our service and the machine*, *and is petrified that when the trial ends that machine will be taken away from him*, *because it has become his lifeline…he’s become dependent and believes he should be getting phone calls on a regular basis* (Health Professional) Fairbrother 2013	*I’m okay at 87% (oxygen saturation) upwards and I never get better than 92*. *Even when I’m very well*, *I never get better than 92*. *But I go out and about and I do what I need to do and I manage it by walking* Fairbrother 2013
*If they take out tomorrow…and I go back to*, *have to rewire the panic button up again*, *you know*, *and that type of thing*, *am I going to be calling the girls [nurses] out more*, *am I going to be in hospital more*, *am I going to go back to square one just because of that equipment* Gale 2013	*It’s made me more confident in myself because I know what’s happening*. *Well*, *before I didn’t*, *did I*?.* *.* *.*If I didn’t have that [telehealth]*, *I wouldn’t know how low I was*, *I’d just carry on with a normal day*, *wouldn’t I*? *Now you’ve got that*, *it puts you aware of what’s happening* Gale 2013
*… But there is not much to having it if it doesn’t go to some facility like it did to [home nurse]*. *So that she could keep track*. Huniche 2013	*Seeing my data on the web portal gives me a better understanding of how to exercise and interpret the development of my symptoms when I experience the onset of an exacerbation* Dinesen 2013
*‘felt it was a waste of time’ ‘ they [respiratory nurses] tell me to do this when I’m not well and they [respiratory nurses] come and see me* Williams 2014	*It’s a presence in the home*, *it encourages me to do what I call breathing exercises* Williams 2014

### 1: The influence of telehealth on the moral dilemmas of help seeking

A persistent theme across five of the seven studies relates to how telehealth eased patient responsibility for seeking help and legitimised contact with health care providers. Patients felt burdened by dilemmas of when to seek help prior to using telehealth; this had often delayed help seeking. Analysis across first and second order constructs identified that telehealth lifted responsibility for help seeking in two ways: 1. By providing patients with ‘concrete evidence’ to contact their health care provider through identification of abnormal readings; 2. by shifting the responsibility for initiating contact to health care providers.

#### Dependency versus self-care

On the whole, studies reported that patients experienced a sense of relief that telehealth legitimised contact with their care providers and helped them feel supported in self-care. However, one study reported health professionals’ concerns that telehealth may promote dependency on healthcare providers and telehealth data, especially in patients with more severe disease [28]. Further analysis of first and second order constructs identified that most papers reported these conflicting consequences of telehealth within 1st order constructs but they were not always recognised or explicitly reported in authors’ 2nd order interpretations. Primary data across five studies indicates telehealth functions between both self-care and health care dependency (see Table 3 for examples). Studies which reported data on patients’ perspectives had a focus on exploring how patients used telehealth in their every-day lives and how it helped them to self-manage [32–35]; therefore, there was an emphasis towards exploring the self-care side of the continuum. For example, the Danish study [32, 33] demonstrates several ways patients used telehealth readings to self-care. This study did report that 5 out of the 22 patients did not make use of telehealth readings [32, 33]. The Williams et al. study [34] also reported how telehealth enabled patients to self-manage; suggesting those who did not experience benefit were less engaged in managing their condition.

### 2: Conflicting consequences of interactional transformation in telehealth

This focuses on telehealth and the consequences of interactional transformation, including the conflict between patients’ and health professionals’ experiences that are engendered by this change in interaction. Telehealth can be seen to transform interactions between patients and their health care providers in three ways, by: 1. Shifting emphasis from traditional face to face consultations to remote consultations; 2. Changing the frequency of interaction between patients and their health care providers and 3. Providing a ‘*benign form of surveillance’* [35] through remote monitoring of physiological and other data.

#### Reassurance

A consistent theme reported in all studies was that telehealth provided patients with a sense of reassurance and a strong sense of feeling ‘looked after’. No studies reported any patient concern regarding the change in mode of interaction, although it is not clear in 6 studies whether significant shifts occurred. Only one study clearly reported that home visits were replaced by remote consultations which patients found suitable [30, 31]. Patients’ sense of reassurance came from two other components of interactional transformation: through an increase in patient-health care provider interaction and through knowing that their data was being remotely monitored. Components of interactional transformation are interlinked as patients valued being remotely monitored but only in the context that if problems occurred they would be able to interact with a human being.

Patients concentrated on, and valued, the increased contact and level of continuity that telehealth enabled. A sense of continuity associated with interventions brought additional reassurance: this was evident within three of the papers [28, 33, 34]. In the Williams et al. study, patients’ sense of reassurance came from the *‘virtual link’* obtained by sharing their data with a research nurse, despite knowing their data was not monitored regularly nor intended to replace usual care. A sense of continuity came from increased telephone contact with telehealth professionals for patients in the Fairbrother et al., 2013 paper; this enabled trusting relationships to be formed, in part because telehealth professionals facilitated patients’ access to other health professionals. Patients in one study [32, 33] reported feeling a *‘sense of security’* due to regular weekly contact with a specialist health care provider and through access to telehealth data.

The exchange of telehealth data between patient and health care provider plays an important role in patients’ sense of reassurance through enhanced feelings of social connectedness. This theme was explicit in two studies: Gale and Sultan study [35] reported that remote monitoring alleviated feelings of isolation and patients felt a connection to their health care providers. Feelings of isolation were reduced in another study [32, 33] through patients and health professionals sharing telehealth data between patients and health professionals which enabled shared decision making.

#### Risk

Reassurance provided through increased social connectedness may be threatened when telehealth does not live up to patients’ expectations: this was evident in the Ure et al. study [36] whereby one man delayed help seeking as he mistakenly believed his heart problem would be identified through telehealth monitoring.

In contrast to the generally positive experience of patients, there is evidence across 3 studies [28–31, 36] that health professionals held less positive views on the interactional changes brought about by telehealth. There was evidence that health professionals perceived telehealth as undermining their capacity for holistic surveillance: they lost the view of the social and physical environmental context. This theme is most prevalent throughout the Mair et al., 2008 paper [30], where regular video-link consultations were used in place of home visits to patients who were experiencing acute exacerbation of COPD. In contrast to patients’ positive views on the acceptability of remote care within this and other studies, a strong theme reported was that nurses felt remote consultations impeded communication with patients and this compromised the nurse-patient relationship. In contrast, health professionals in another study [29] felt telehealth helped to form trusting patient relationships due to an increase in the frequency of interaction; this was perhaps due to telehealth monitoring patients over a 12 month period, whilst telehealth was in place for only 3 weeks in the Mair et al./Hibbert et al. study [30, 31]. However, as identified in the construct above, health professionals in the Fairbrother et al. study [28, 29] expressed concern that increased interaction plus monitoring created dependency in patients as well as increasing their workload (this will be explored in third order construct 3).

Safety issues were raised by health professionals in 3 studies [28–31, 36]. Nurses in the Mair et al./Hibbert et al. study [30, 31] felt that remote interaction resulted in less accurate clinical assessments, in part due to the technical issues that they encountered with the equipment: nurses questioned the safety of telehealth and concluded it was unsuitable in their care context. In 2 studies [28, 29, 36] health professionals expressed concern that telehealth may lead to an overtreatment of COPD. Telehealth data needs to be assessed accurately within the overall context of the individual patient’s condition. Telehealth therefore requires health professionals to work in new ways: this necessitates a change in the type of interactions that occur and transition to a more technical type of working. This theme will be explored in further detail below.

### 3: Telehealth as transforming the nature of work and the consequences for identity and burden

Telehealth can be seen to transform the nature of work for both patients and health professionals. Using telehealth reconfigures patients as more active members of the workforce; for the most part, patients welcome this. New responsibilities occur as patients now play a more ‘active’ role in their care by taking on monitoring of their physiological signs and becoming more involved in managing their condition; this can bring welcomed responsibility but also burden.

#### Empowerment

Only one study [34] identified patients’ hesitancy in taking on the burden of monitoring prior to telehealth being installed. However, patients’ initial concerns were not realised as they found the equipment easy to use. This resonates with the majority of studies that identified patients’ acceptability of performing these new roles, with most patients finding the equipment easy to use [28–33, 35, 36]. Patients were aware that they were taking on roles traditionally performed by health professionals and this gave them a sense of accomplishment. There is evidence that these new responsibilities are not performed by patients alone but also by family members who become more actively involved in the management of the patients’ condition through the use of telehealth [30, 32, 33].

#### Burden

Patients often viewed becoming a more active member of the workforce as a positive consequence of using telehealth and they reported feelings of empowerment and independence in being able to better manage their condition. Whilst most patients welcomed this new way of working, some patients can feel over burdened by the responsibility: when readings are abnormal or when they deem telehealth equipment too difficult to use [27, 30, 32, 33, 36]. For example, two studies reported patient anxiety when readings breached agreed ‘normal’ parameters [27, 32, 33]. No studies explored this in any depth so there was no insight into the types of people who may feel burdened by knowledge of [abnormal] physiological readings [31]. In the two studies that tested telehealth in acute exacerbation of COPD [27, 30, 31], health professionals questioned patients’ ability to perform tasks and/or felt telehealth would be too bothersome for them; they felt it particularly unsuitable for older people. In one study, this influenced their decision to offer telehealth; in part, this also stemmed from their perception of the [lack of] user friendliness of the equipment [27]. Equipment failure was an issue in most studies [27–31, 33, 36]. Whilst studies reported mainly patients’ positive views of telehealth, despite equipment failure, it was reported that a minority of patients stopped using telehealth due to dissatisfaction with equipment [27, 36]. This was not an issue explored in any detail because patients who withdrew from using telehealth did not appear to have been interviewed in the qualitative studies.

Less positive views were reported by health professionals who indicated that the technical type of work brought about by telehealth increases burden and undermines aspects of their professional identity. All studies that investigated health professionals’ experiences reported telehealth increased health professional burden by increasing their workload [27–31, 36]. In one study [30, 31] nurses were expected to install the equipment in patients’ homes which they deemed inappropriate; problems with connectivity, failed equipment and incompatibility of equipment with existing NHS systems also increased workload [29–31]. Furthermore, health professionals reported making more phone calls or home visits to determine patients’ health status after receiving ‘abnormal’ readings [36].

Burden also comes from health professionals feeling that a shift to a more technical type of work undermines their professional identity. In one study [30], specialist respiratory nurses were concerned that increased telehealth use could lead to a decrease in the number of nurses needed; moreover, they felt telehealth threatened the very aspects of their role which were integral to their identity as a specialist respiratory nurse.

### Summary of synthesis

**Fig 2:**
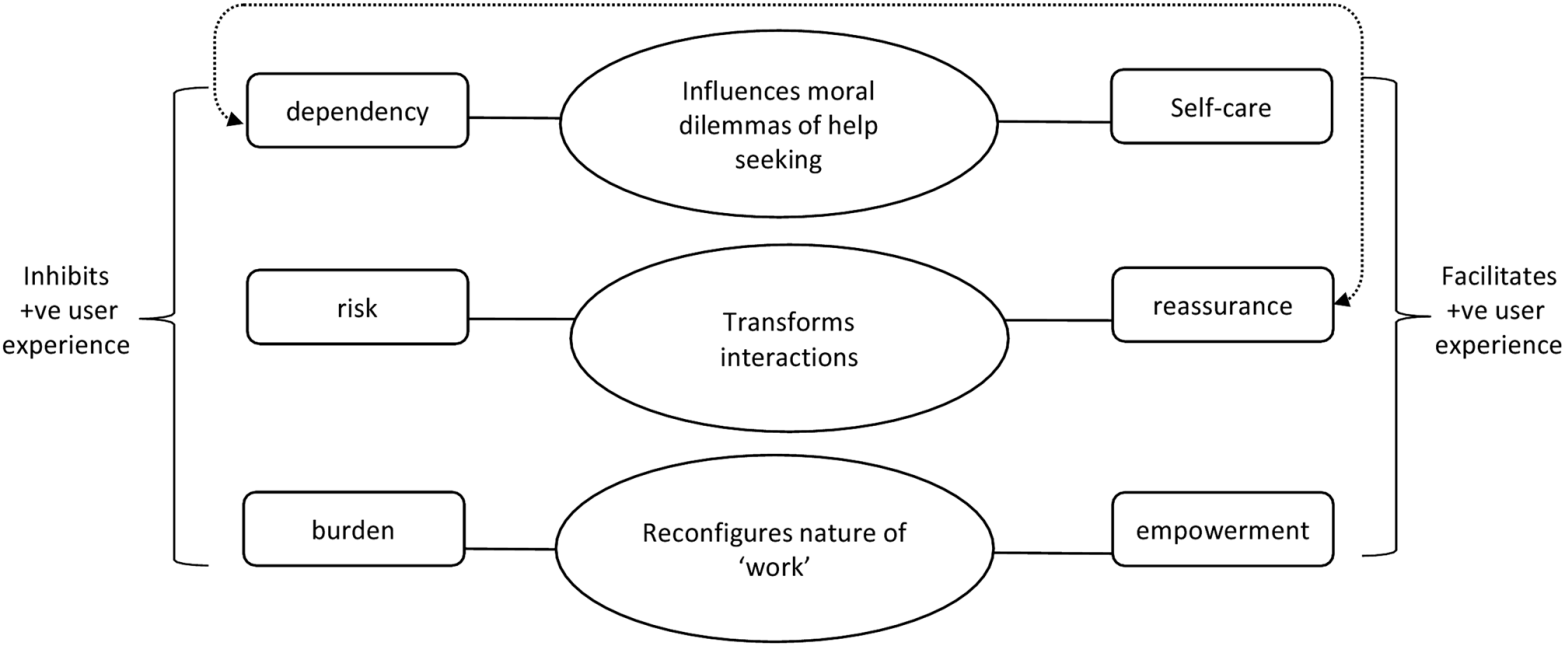
Theoretical framework to show conflicting consequences of telehealth use in COPD.

It was apparent from the second order constructs within the included studies that the authors of most papers had identified both positive and negative experiences of telehealth use within the management of COPD; in particular, where both patient and health professional views were sought, they identified an incongruence of views between patients and health professionals (patients on the whole described more positive experiences). By combining results from across all included studies and applying a ‘line of argument’ synthesis, we were able to derive new insights from the data through conceptualising these contrasting experiences. We found that instead of there being independent barriers and facilitators to telehealth use, users perceive telehealth as having the same characteristics; however, these can be viewed positively or negatively depending on users’ experiences. This enabled us to develop a theoretical framework in order to explicitly identify the conflicting consequences of telehealth use in COPD (see Fig 2). The framework centres on three third order constructs which have the potential to inhibit *or* facilitate positive user experience: *the influence on moral dilemmas of help seeking* (which is perceived to enable dependency *or* self-care); *transforming interactions* (perceived to increase risk *or* reassurance); and *reconfiguration of ‘work’ practices* (perceived to cause burden *or* empowerment).

Telehealth took away the moral dilemma of help seeking for patients as telehealth enabled them to contact health professionals and/or receive greater input when telehealth triggered health professionals to contact them. Health professionals appear to perceive this as indicative of creating greater dependency in patients, whereas most patients in the studies view this in terms of providing greater support to enable them to self-care. Synthesis of the included studies revealed that telehealth functions on a continuum between increasing dependency and increasing self-care. It was not clear from the studies if the same patients function between dependency and self-care—at times needing more input from their healthcare provider during their illness trajectory or whether certain types of patients who have the potential to self-care are made more dependent by telehealth; more research is needed into this.

The characteristic of *transforming interactions* threatened positive user experience it if was perceived to increase risks to patient safety. For example, if it was portrayed as impeding clinical assessment or when over reliance on telehealth as a monitoring device led to a false sense of security. Conversely, *transforming interactions* enables positive user experience when it is perceived to increase social connectedness and this together with having readings remotely monitored, increases feelings of reassurance. However, there is a tension when ‘positive’ feelings of reassurance are perceived to derive from telehealth enabling greater dependency on health care providers.

Telehealth as *reconfiguring the nature of ‘work’* for both patient and health professionals is perceived to create burden when it increases patient anxiety, increases workload or threatens the professional identify of health professionals. Patients perceive this new way of working as a positive experience when it empowers them to become more independent in their ability to self-care.

## Discussion

In attempting to introduce telehealth technologies into COPD services, the main focus has been upon reducing the demand for health care resources and much less attention has been paid to user experience. This synthesis shows that telehealth technologies have the potential to be beneficial in the management of COPD compared to usual care alone (such as enabling self-care, providing feelings of reassurance and empowerment) but highlights how these benefits can each be viewed as detriments (increasing dependency, risk, and burden). Therefore, a key strength of this synthesis has been to bring together the perspectives of different users (patients, their carers and health professionals); this enabled us to develop our conceptual framework that accentuates the positive *and* negative dimensions of telehealth use. The synthesis did not show that the *type* of telehealth intervention implemented was related to user acceptability or its ability to increase self-management; however, this warrants further investigation as we suspect differences may exist between telehealth interventions that are more likely to enable self-management and increase user acceptability.

This synthesis highlights a notable difference between patients’ and health professionals’ views and experiences of telehealth, with patients being generally more positive about telehealth use than health professionals. Reasons for this may be because the majority of patients included in the sample received enhanced care as telehealth was an addition to usual care in the majority of studies: less positive views may have been expressed if telehealth had replaced existing services. In addition, it appears that the only patients who were included in the studies were those that had already agreed to and were using telehealth technologies; patients who had refused telehealth or those that withdrew from the intervention do not seem to have been included in any of the studies (this is discussed further in the limitations section below). In contrast, health professionals (particularly nurses working at the point of delivering care) are often removed from the decision making process to incorporate telehealth into their current service provision; plus, telehealth within the included studies seemed to operate outside of existing service provision.

Whilst our synthesis highlights mainly negative health professional perceptions of telehealth, a qualitative study which was part of the Whole System Demonstrator trial highlighted mixed perceptions from front line health professionals delivering telehealth services to patients with heart failure, COPD and diabetes [37]. Their findings support our interpretation that telehealth can be perceived either to burden *or* empower which leads to dependency *or* self-care. Nurses (who were actively engaged in providing care via telehealth) generally perceived telehealth as empowering patients by increasing their understanding of their disease and helping them to change health behaviours, whilst GPs (who were less engaged in providing care via telehealth) generally perceived telehealth as a burden on patients: provoking anxiety in ‘well’ patients through clinical surveillance and overburdening patients with severe disease. Furthermore, there was evidence from nurses that telehealth had enabled self-care but both nurses and GPs had concerns regarding patients becoming dependent on the technology. GPs were also more likely to perceive telehealth as a burden in terms of increasing their workload whilst nurses mainly welcomed its introduction provided it supported but did not replace traditional care [37]. Our synthesis highlights the threat to identity and professional roles that nurses can feel when their traditional roles are changed to a more technical type of work. Building on from this, it is also important to understand and acknowledge the potential conflict between professional roles that may occur when telehealth is introduced in to existing primary care services. The Fairbrother et al. study [28] alluded to this, whereby health professionals were divided in their views on whether cross-boundary working had been successful, although this was not the focus of the study. A previous study has also highlighted the tensions that existed between telehealth service (THS) professionals (who were involved in delivering a telephone advice service for patients with a LTC) and practice nurses and GPs (who were not involved in the delivery of telehealth) [38]: not only were GPs and practice nurses sceptical regarding the benefits of telehealth in LTC management but there was evidence that nurses and GPs perceived telehealth to threaten their existing roles and this brought about tensions regarding professional boundaries. When telehealth services are implemented, care needs to be taken to recognise how telehealth impinges upon professionals’ understanding of their roles and identity [38]. This highlights the importance of involving health professionals (including those at the point of delivering care) in the decision making process of changing services/developing new services from the outset.

Four of the seven studies intimated that the telehealth interventions tested were unlikely to be sustained into routine care [27, 29, 30, 35]. This is a common problem of telehealth studies, in that the interventions are only sustained into practice for as long as the research study lasts. One reason for this may be that telehealth technologies are often evaluated using pre-determined outcomes, which often relate to current policy goals (such as reducing resource demands); a problem arises when pre-determined outcomes are not met, but other, arguably equally important, goals are met [39]. For example, previous evidence suggests that the stigma of having COPD, together with feeling responsible for their illness due to their smoking habits, contribute to some people’s reluctance to seek help [40]. Our synthesis highlights how telehealth interventions met a previously unmet need by increasing access to and legitimising contact with health professionals. So, the pre-determined goals of telehealth (e.g. to realise efficiency savings) may need to be changed or adapted as the technology develops, rather than halting implementation because pre-determined goals have not been met [39]. This is difficult to achieve within the confines of an RCT; yet to date there has been a reliance on conducting RCTs for evaluating telehealth; these can impede implementation because the RCTs conducted may bear little resemblance to what happens in the real world of healthcare [41]. Therefore, despite RCTs being classed as the ‘gold standard’ of research, some have questioned the usefulness of them when evaluating something as complex as a telehealth intervention [41–43].

In appraising the quality of studies included in the synthesis, we chose to use a modified version of the CASP appraisal tool. This was a useful tool to appraise conduct and rigour [22] but a limitation of the CASP tool is that it does not differentiate between studies which are conceptually rich compared to those which are descriptive [15, 22]. Therefore, whilst the CASP checklist helped to guide our appraisal of quality in the included studies, in reality, we also used our experience as qualitative researchers to judge how far each paper contributed to the overall synthesis [22].

### Limitations

As we only included studies that were published in the English language, for resource reasons, relevant studies may have been excluded from this review. The majority of studies included (n = 6) were based in the UK, with only one study being based in Denmark; therefore, the findings may not translate to other countries. The study may be viewed to be methodologically weakened as we did not perform double screening of references; however, a subset of references (10 percent) were screened by a second author, with high inter-rater reliability observed, suggesting satisfactory screening methods were employed. We only included studies that conformed to our definition of telehealth; this may be considered a narrow definition but our interest was in interventions where patients played an ‘active’ role in their care (i.e. through having to take their physiological readings or answer symptom questionnaires) so that we were not including studies where the same care was being delivered but remotely (i.e. telephone consultations). None of the included studies appeared to include patients who had refused telehealth and/or those who had withdrawn from the intervention/study. This can inevitably introduce bias into the sample and could explain the overwhelmingly positive views of the patients within the included studies. This is an important limitation of the findings because a recent mixed methods systematic review assessing the uptake and sustained use of telehealth in trials of COPD and heart failure patients found that on average 32% of patients refused telehealth (refusal rates ranged from 4–71%), with 20% of those who agreed to participate withdrawing from the studies (withdrawal rates ranged from 4–55%) [44]. The main reasons given for refusing telehealth were patients’ disinterest in the technology and no felt need for telehealth whilst the main reasons for withdrawal were patients not wanting to use equipment, deteriorating health and technical problems [44]. Our search found only one study (which was conducted by one of our authors—CS) [45] which qualitatively explored patients’ reasons for non-participation in a telehealth trial. This study was excluded from the review because it was a mixed sample containing COPD, diabetic and coronary heart disease patients and the results were not separated. The study conducted in-depth interviews with 22 people who had either declined or withdrawn from a telehealth trial, together with 23 observational home visits. They identified the following themes for why participants refused telehealth: patients’ perceived a lack of technical competence and a perceived inability to use the equipment; they perceived telehealth as a threat to their identity and independence; satisfaction with current services and an expectation that telehealth may lead to disruption of these services [45]. Patients withdrew from telehealth mainly due to technical difficulties with the equipment and frustration with slow response to technical problems [45].

### Implications for future research

The theoretical framework developed is an early attempt at an explanatory model of the consequences of telehealth use as perceived by patients and their carers (informal and health professionals). Further qualitative research is needed to understand the key ingredients that will facilitate positive user experience of telehealth (i.e. increase self-care not dependency; enable feelings of reassurance not risk; ensure feelings of empowerment not burden). Whilst this synthesis has highlighted the potential for telehealth to be successful in the management of COPD, further qualitative research (through engagement with all potential users of telehealth) needs to establish whereabouts in the illness trajectory telehealth would be most useful. For example, from our synthesis we have highlighted that health professionals in two studies deemed telehealth to be unsuitable for patients with acute exacerbation of COPD, although patients accepted telehealth as an acceptable mode of care [30]. Future research also needs to establish what types of patients with COPD may benefit from telehealth. Future research should also explore with health professionals, the change in clinical role to a more technical type of work and/or the potential changes to delineation of roles when telehealth is introduced. More collaborative research is needed to bridge the gap between technological innovation and successful service delivery; this could involve research that engages industry with patients, their carers and health professionals.

## Conclusion

Through conducting a meta-synthesis on the qualitative literature available, we have been able to conceptualise user experience to identify the factors which inhibit or promote positive user experience of telehealth; this has implications for the future design and successful implementation of telehealth services. Further qualitative research, which includes [potential] users of telehealth is required at an earlier stage of technology and service development.

## Supporting Information

S1 File Ovid Medline search strategy. (DOCX)

S2 File Quality Appraisal using adapted version of CASP tool. (XLSX)

S3 File PRISMA checklist. (DOC)
