# Supplementary material for: The Contradictions of Telehealth User Experience in Chronic Obstructive Pulmonary Disease (COPD): A Qualitative Meta-Synthesis
Source: PLoS One. 2015 Oct 14;10(10):e0139561. doi: 10.1371/journal.pone.0139561 (PMC4605508; doi:10.1371/journal.pone.0139561)
Supplement: S1 File — (DOCX) [file pone.0139561.s001.docx]

| **Ovid Medline Search conducted on 04.10.14** | | | |
| --- | --- | --- | --- |
| **#** | **Searches** | **Results** | **Search Type** |
| 1 | telehealth.af. | 1655 | advanced |
| 2 | (telecare or tele-care).af. | 2324 | advanced |
| 3 | (telemedicine or tele-medicine).af. | 14877 | advanced |
| 4 | (mhealth or m-health).af. | 3305 | advanced |
| 5 | telehomecare.af. | 60 | advanced |
| 6 | telehealthcare.af. | 54 | advanced |
| 7 | (telemonitoring or tele-monitoring).af. | 628 | advanced |
| 8 | 1 or 2 or 3 or 4 or 5 or 6 or 7 | 18527 | advanced |
| 9 | exp Pulmonary Disease, Chronic Obstructive/ | 37419 | advanced |
| 10 | chronic obstructive pulmonary disease.mp. [mp=title, abstract, original title, name of substance word, subject heading word, keyword heading word, protocol supplementary concept word, rare disease supplementary concept word, unique identifier] | 26444 | advanced |
| 11 | chronic obstructive airway* disease.mp. [mp=title, abstract, original title, name of substance word, subject heading word, keyword heading word, protocol supplementary concept word, rare disease supplementary concept word, unique identifier] | 655 | advanced |
| 12 | exp Lung Diseases, Obstructive/ | 172381 | advanced |
| 13 | emphysema.mp. [mp=title, abstract, original title, name of substance word, subject heading word, keyword heading word, protocol supplementary concept word, rare disease supplementary concept word, unique identifier] | 27376 | advanced |
| 14 | exp Bronchitis/ | 26680 | advanced |
| 15 | bronchitis.mp. [mp=title, abstract, original title, name of substance word, subject heading word, keyword heading word, protocol supplementary concept word, rare disease supplementary concept word, unique identifier] | 28261 | advanced |
| 16 | exp Emphysema/ | 10971 | advanced |
| 17 | 9 or 10 or 11 or 12 or 13 or 14 or 15 or 16 | 197171 |  |
| 18 | 8 and 17 | 312 | advanced |

S1 File. Ovid Medline Search Strategy
